# ADMM-EM Method for *L*_1_-Norm Regularized Weighted Least Squares PET Reconstruction

**DOI:** 10.1155/2016/6458289

**Published:** 2016-10-19

**Authors:** Yueyang Teng, Hang Sun, Chen Guo, Yan Kang

**Affiliations:** ^1^Sino-Dutch Biomedical and Information Engineering School, Northeastern University, Shenyang 110004, China; ^2^Shenyang Branch of Philips Healthcare (Suzhou) Co., Ltd., Shenyang 110004, China

## Abstract

The *L*_1_-norm regularization is usually used in positron emission tomography (PET) reconstruction to suppress noise artifacts while preserving edges. The alternating direction method of multipliers (ADMM) is proven to be effective for solving this problem. It sequentially updates the additional variables, image pixels, and Lagrangian multipliers. Difficulties lie in obtaining a nonnegative update of the image. And classic ADMM requires updating the image by greedy iteration to minimize the cost function, which is computationally expensive. In this paper, we consider a specific application of ADMM to the *L*_1_-norm regularized weighted least squares PET reconstruction problem. Main contribution is derivation of a new approach to iteratively and monotonically update the image while self-constraining in the nonnegativity region and the absence of a predetermined step size. We give a rigorous convergence proof on the quadratic subproblem of the ADMM algorithm considered in the paper. A simplified version is also developed by replacing the minima of the image-related cost function by one iteration that only decreases it. The experimental results show that the proposed algorithm with greedy iterations provides a faster convergence than other commonly used methods. Furthermore, the simplified version gives a comparable reconstructed result with far lower computational costs.

## 1. Introduction

Positron emission tomography (PET) is an important imaging tool in modern medicine and provides noninvasive quantification of the biochemical and biological processes inside living subjects. Several reconstruction methods have been developed and applied in clinical practice. These methods can be roughly divided into two categories: analytical methods and iterative methods. Iterative methods have attracted more attention because they generally model imaging physics better and are more capable of suppressing noise artifacts than analytical methods. A basic target of PET reconstruction is to solve a system of the following form:

(1) $$\begin{array}{c} Y=PX+S, \end{array}$$

where *X* represents the biochemical activity distribution inside a subject, *Y* denotes the measured projections, *S* is scatter and random events, and *P* is a predetermined system matrix, where *X* ∈ *R* ^*N*×1^, *Y* ∈ *R* ^*M*×1^, *S* ∈ *R* ^*M*×1^, and *P* ∈ *R* ^*M*×*N*^. All of these are fully nonnegative.

A classical approach is to select *X* such that the weighted least squares (WLS) error [1] between *Y* and *PX* + *S* is minimized:

(2) $$\begin{array}{c} \underset{X\geq 0}{\min}\;F\left(\;X\;\right)={\left\|\;PX+S-Y\;\right\|}_{\Sigma }^{2}. \end{array}$$

Σ is a diagonal matrix with the diagonal element Σ_*ii*_ = 1/*σ*_*i*_, where *σ*_*i*_ is the variance of the *i*th measurement. In theory, the values in *Y* are larger than those in *S*; however, in practical applications, the latter one could exceed the former one when estimating *S*.

Although an iterative algorithm can more effectively suppress noise propagation than conventional filtered back-projection, it may fail in special cases, such as increased amount of noise, sparse projections, or low dose (which results in high noise or poor SNR). In fact, the problem of reconstructing PET image data is an ill-posed inverse problem. Over the past twenty years, efforts have been made to solve these problems by employing regularization techniques [2–8]. A general method is to introduce* a priori* knowledge to constrain the solution space, which can be expressed as a regularization (or penalization) on the reconstructed image to reflect information on the properties of acceptable images. Tikhonov regularization [9, 10] is a popular method that generally leads to a unique solution. There are many methods for solving such a quadratic programming problem [1, 11]. However, they exhibit a weak ability to preserve the edges while smoothing the interior of the image.

Another viable regularization method is the *L*_1_-norm regularization [12–16], in which one seeks to find the solution of the following optimization problem:

(3) minX≥0 FX+βRX1,

where *β* > 0 serves as a penalty parameter and *R* is a linear operator (e.g., gradient operator and orthonormal transformation). The penalty parameter *β* controls the tradeoff between data fidelity and resolution (image smoothness). Several linear operators *R* have been proposed, such as the first- or second-order derivative or wavelet basis. Note that *R* could include negative elements. The underlying philosophy in working with *L*_1_-norm regularization is that most images have a sparse representation [17].

In recent years, the subgradient-based method [18] has been developed for solving convex and nonconvex optimization problems; this method takes a subgradient related surrogate function at each step to obtain the update. Another method is the alternating direction method of multipliers (ADMM) [19, 20]. ADMM decomposes the original problem into three subproblems, and then it sequentially solves these subproblems at each iteration. For medical imaging, ADMM will distributively minimize the augmented Lagrangian-related function to solve for the additional and primal variables (pixels), and then it updates the dual variables, which are associated with a coupling constraint.

One can reformulate the optimization problem in (3) by imposing the extra constraint *V* = *RX*, which leads to the following optimization problem:

(4) min FX+βV1s.t. X≥0, V=RX.

Note that (3) and (4) have the same solution. The scaled augmented Lagrangian function [19] is introduced to overcome these difficulties, and it is defined as follows:

(5) LX,V,μ=FX+βV1+ρ2RX−V+μ22−ρ2μ22,

where *μ* is the dual variable or Lagrange multiplier and *ρ* > 0 is the penalty parameter. When *ρ* = 0, the augmented Lagrangian can be reduced to the unaugmented (common) version.

By applying distribution optimization for *V* and *X* and dual ascent to *μ*, a unified framework can be introduced to solve the *L*_1_-norm regularized WLS reconstruction problems. For the *V*-update, one exploits the separability *L*(*X* ^*t*^, *V*, *μ* ^*t*^) in *V*, that is, *L*(*X* ^*t*^, *V*, *μ* ^*t*^) = ∑_*i*=1_^*M*^ *L*_*i*_(*X* ^*t*^, *V*_*i*_, *μ* ^*t*^), to solve for each *V*_*i*_ independently. A solution can be found in [17], which is the well-known shrinkage method. To update *μ*, a simple gradient-ascent method can be used. When not considering the details of the *X*-update, the ADMM's framework can be formulated [19] as follows.

**Algorithm 1 (ADMM general framework).**

One has

(1) *V*_*i*_^*t*+1^ = (|(*RX* ^*t*^ + *μ* ^*t*^)_*i*_| − *β*/*ρ*)_+_sgn⁡[(*RX* ^*t*^ + *μ* ^*t*^)_*i*_], *i* = 1,…, *M*;
(2) *X* ^*t*+1^ = arg⁡min⁡_*X*≥0_⁡*L*(*X*, *V* ^*t*+1^, *μ* ^*t*^);
(3) *μ* ^*t*+1^ = *μ* ^*t*^ + *RX* ^*t*+1^ − *V* ^*t*+1^.

Here, the *X*-update is a difficult problem that minimizes the following function with the nonnegativity constraint:

(6) $$\begin{array}{c} L\left(\;X,V^{t+1},\mu^{t}\;\right)=F\left(\;X\;\right)+\left(\;\frac{\rho }{2}\;\right){\left\|\;RX-V^{t+1}+\mu^{t}\;\right\|}_{2}^{2}. \end{array}$$

When 2*P* ^*T*^ *P* + *ρR* ^*T*^ *R* is invertible, we may obtain a unique global solution, which has been used in [21, 22].

(7) X=2PTP+ρRTR−12PTY−S+ρRTVt+1−μtX=max⁡0,X,

where the truncation below zero is necessary for constraining the solution to the nonnegative space. This formulation is feasible in theory if the regularization process guarantees the nonsingularity of the matrix.

A viable method is the gradient-based method. The steepest descent method is perhaps the simplest technique to implement, which takes the negative gradient as the descent direction:

(8) Xt+1=Xt−αt∇LXt,Vt+1,μtXt+1=max⁡Xt+1,0,

where the superscript *t* denotes the *t*th iteration and *α*(*t*) is the step size. Of course, negative pixel values still need to be truncated. The convergence of the gradient-based methods depends on the choice of step size, which is problematic for practical implementations.

Let *α*(*t*) = *α* be a constant, where 0 < *α* < 2/‖∇^2^ *L*_*X*_(*X* ^*t*^, *V* ^*t*+1^, *μ* ^*t*^)‖ (‖·‖ denotes the maximum eigenvalue); then (8) becomes the projected Landweber method [23]:

(9) Xt+1=Xt−α∇LXt,Vt+1,μtXt+1=max⁡Xt+1,0.

The conjugate gradient method is a popular approach, which is often implemented as an iterative algorithm applicable to sparse systems for large-scale problems.

However, for large-scale problems such as PET reconstruction, the inverse matrix method becomes very costly from a computational perspective. Furthermore, the “pure” steepest descent method and the “pure” conjugate gradient method do not meet the nonnegativity constraint, and consequently, negative pixel values need to be truncated. The truncation, however, leads to a divergent modification. The projected Landweber method, however, is able to meet the nonnegativity, but its convergence cannot be proven theoretically. In addition, the projected algorithms also destroy the monotonic decreasing properties of the decomposed cost function.

In fact, ADMM is a framework of distribution optimization, which is not limited to PET reconstruction with the nonnegativity constraints. With different constraints and distributed variables, ADMM can be applicable in many other fields, such as multiple-block convex programming [24], ADMM for tomography with nonlocal regularizers [25], linear classification [26], and optimal power flow problems [27].

In this paper, we consider a specific application of ADMM to the *L*_1_-norm regularized WLS reconstruction problems. Here, we do not change the framework of ADMM; rather, we develop a new algorithm for PET image reconstruction. The proposed approach is applicable to several medical image reconstruction problems, such as TV regularized [13] and wavelet regularized [14] image reconstruction. We focus on a key subproblem: the *X*-update. A multiplicative update rule is derived to iteratively and monotonically (in the sense of decreasing cost function) update the pixel values. Similar to the EM algorithm [28, 29], the pixel-update algorithm also intrinsically satisfies the automatic satisfaction of the nonnegativity constraint without the need for an adjustable step size. We provide a rigorous convergence proof for the proposed *X*-update, which shows that the algorithm will iteratively pursue a single global optimum. The *X*-update that optimizes the subproblem inevitably leads to high computational costs, and we can replace it by a single iteration algorithm to decrease the decomposed cost function of the subproblem, which is an often used strategy in distributed optimization. The experimental results demonstrate that the proposed algorithm (with greedy reconstruction of the pixels) provides better performance compared to those of other commonly used methods with respect to image qualification and convergence speed. The results also show that the simplified version provides a comparable reconstructed result but at a considerably lower computational cost compared to the existing methods.

## 2. Methodology

For notational simplicity, we define *C* = −*V* ^*t*+1^ + *μ* ^*t*^ as a constant vector, and then we define the cost function in (6) as follows:

(10) ΦX=LX,Vt+1,μt=FX+ρ2GX,where:  GX=RX+C22.

We will solve this optimization problem using a modified EM-type algorithm. As mentioned in many articles [30–33], a surrogate function, as defined below, is useful in algorithm derivation and convergence proof.

**Definition 2 (surrogate).**

The function *ψ*(*X*∣*X* ^*t*^) is a surrogate of Ψ(*X*) at *X* ^*t*^ (fixed) if *ψ*(*X* ^*t*^∣*X* ^*t*^) = Ψ(*X* ^*t*^) and *ψ*(*X*∣*X* ^*t*^) ≥ Ψ(*X*).

Clearly, Ψ(*X*) is decreasing under the update *X* ^*t*+1^ = min_*X*_ *ψ*(*X*∣*X* ^*t*^) because of

(11) $$\begin{array}{c} \Psi \left(\;X^{t+1}\;\right)\leq \psi \left(\;X^{t+1}\;|\;X^{t}\;\right)\leq \psi \left(\;X^{t}\;|\;X^{t}\;\right)=\Psi \left(\;X^{t}\;\right). \end{array}$$

There are two important properties for the surrogate: additivity and transitivity. For the former, the sum of two surrogates is a surrogate of the sum of two original functions. For the latter, the surrogate of the surrogate of a function is a surrogate of this function. Following these properties, we will construct the surrogates for *F*(*X*) and *G*(*X*).

### 2.1. Surrogate for *F*(*X*)

We construct a surrogate *f*(*X*∣*X* ^*t*^) by the convexity. Let

(12) λi∗=SiPXt+Si,λij=PijXjtPXt+Si

that satisfy *λ*_*i∗*_, *λ*_*ij*_ ≥ 0 and *λ*_*i∗*_ + ∑_*j*=1_^*N*^ *λ*_*ij*_ = 1. They can be the convex combination coefficients such that

(13) fX ∣ Xt=∑i=1Mσiλi∗Siλi∗−Yi2+∑j=1NλijPijXjλij−Yi2.

It can be verified that *f*(*X* ^*t*^∣*X* ^*t*^) = *F*(*X* ^*t*^). When considering Jensen's inequality and the convex combination coefficients *λ*_*ij*_, then *f*(*X*∣*X* ^*t*^) ≥ *F*(*X*) is proven by the following inequality:

(14) λi∗Siλi∗−Yi2+∑j=1NλijPijXjλij−Yi2≥PXi−Yi+Si2.

A similar derivation can be found in [34]. Note that *λ*_*ij*_^*∗*^ only relates to the constant term with regard to *X* and can therefore be ignored when minimizing the function *f*(*X*∣*X* ^*t*^).

### 2.2. Surrogate for *G*(*X*)

Since there may be some negative values in the matrix *R*, it is difficult to directly construct a surrogate as above. Some previous works, such as [35, 36], are unable to solve the problem because they fail to guarantee nonnegativity during the iterations, for which we provided a counterexample in [11]. We will utilize an intermediate surrogate to solve the problem. Let $R=\bar{R}-\hat{R}$ and $C=\bar{C}-\hat{C}$, where $\bar{R}$, $\hat{R}$, $\bar{C}$, and $\hat{C}$ are matrices or vectors with nonnegative entries. Subsequently, we can construct a surrogate for *G*(*X*) at *X* ^*t*^.

(15) $$\begin{array}{c} g_{\text{mid}}\left(\;X\;|\;X^{t}\;\right)=\bar{g}\left(\;X\;|\;X^{t}\;\right)+\hat{g}\left(\;X\;|\;X^{t}\;\right), \end{array}$$

where

(16) g¯X ∣ Xt=122R¯X+C¯−R¯Xt+C¯−R^Xt+C^22g^X ∣ Xt=122R^X+C^−R¯Xt+C¯−R^Xt+C^22.

It can be verified that *g*_mid_(*X* ^*t*^∣*X* ^*t*^) = *G*(*X* ^*t*^). By the convexity of *G*(*X*), we view 1/2 as the combination coefficients, leading to

(17) gmidX ∣ Xt=122R¯X+C¯−R¯Xt+C¯−R^Xt+C^22+12−2R^X+C^+R¯Xt+C¯+R^Xt+C^22≥GX.

Following the same process as in Section 2.1, we can construct surrogates $\bar{\bar{g}}(X|X^{t})$ and $\hat{\hat{g}}(X|X^{t})$ for $\bar{g}(X|X^{t})$ and $\hat{g}(X|X^{t})$, respectively. Let

(18) λ¯ij=R¯ijXjtR¯Xt+C¯iλ^ij=R^ijXjtR^Xt+C^i;

then

(19) g¯¯X ∣ Xt=12∑i=1M∑j=1Nλ¯ij2R¯ijXjλ¯ij−R¯+R^Xt−C¯−C^j2g^^X ∣ Xt=12∑i=1M∑j=1Nλ^ij2R^ijXjλ^ij−R¯+R^Xt−C¯−C^j2.

Note that ${\bar{\lambda }}_{ij}^{*}$ and ${\hat{\lambda }}_{ij}^{*}$ are relative to the constant terms and can safely be ignored. Now, we can obtain a surrogate for *G*(*X*) at *X* ^*t*^.

(20) $$\begin{array}{c} g\left(\;X\;|\;X^{t}\;\right)=\bar{\bar{g}}\left(\;X\;|\;X^{t}\;\right)+\hat{\hat{g}}\left(\;X\;|\;X^{t}\;\right). \end{array}$$

### 2.3. Multiplicative Update Rule

We minimize *ϕ*(*X*∣*X* ^*t*^) = *f*(*X*∣*X* ^*t*^)+(*ρ*/2)*g*(*X*∣*X* ^*t*^) to obtain a new iteration. Taking the partial derivatives for *f*(*X*∣*X* ^*t*^), $\bar{g}(X|X^{t})$ and $\hat{g}(X|X^{t})$ leads to

(21) ∂fX ∣ Xt∂Xj=2PTΣPXt+SjXjtXj−2PTΣYj∂g¯¯X ∣ Xt∂Xj=4R¯TR¯Xt+C¯jXjtXj−2R¯TR¯+R^Xt+C¯+C^j∂g^^X ∣ Xt∂Xj=4R^TR^Xt+C^jXjtXj−2R^TR¯+R^Xt+C¯+C^j.

Solving the one-dimensional equations ∂*ϕ*(*X*∣*X* ^*t*^)/∂*X*_*j*_ = 0 leads to a multiplicative update rule, which is the main result in this paper.

(22) Xjt+1=XjtA1+ρ/2A2jA3+ρA4j,where  A1=PTΣY,  A2=R¯+R^TR¯+R^Xt+R¯+R^TC¯+C^,  A3=PTΣPXt+S,  A4=R¯TR¯+R^TR^Xt+R¯TC¯+R^TC^.

The update rule results from the current image multiplied by a factor and is flexible and easy to implement. The derivation process can also be explained in terms of EM optimization: when considering the surrogate as the minimal conditional expectation, its minimization is equivalent to the maximization of the conditional expectation. The algorithm shows two important properties: the iterations are positive if the initial estimate is positive, and the cost function monotonically decreases. For this derivation process, the key step is to replace the minimization of the cost function by minimizing at each iteration the surrogate whose variables are separable. Moreover, the minimization of the surrogate ensures that the cost function decreases.

### 2.4. Specific ADMM and Simplified Version

Now, we can present a specific ADMM that is flexible and convenient for PET reconstruction.

**Algorithm 3 (specific ADMM).**

Given *β* > 0 and *ρ* > 0, then

(1) *V*_*i*_^*t*+1^ = (|(*RX* ^*t*^ + *μ* ^*t*^)_*i*_| − *β*/*ρ*)_+_sgn⁡[(*RX* ^*t*^ + *μ* ^*t*^)_*i*_], *i* = 1,…, *M*;
(2) iteratively update *X* by (22) until some stop rule is satisfied;
(3) *μ* ^*t*+1^ = *μ* ^*t*^ + *RX* ^*t*+1^ − *V* ^*t*+1^.

Note that, in Algorithm 3, ADMM requires greedy iterations to obtain the optimal solution with respect to *X*, which is an expensive operation. In general, an update that decreases the primal cost function is sufficient for use in practical applications; thus, we will provide the following simplified algorithm.

**Algorithm 4 (simplified ADMM).**

Given *β* > 0 and *ρ* > 0, then

(1) *V*_*i*_^*t*+1^ = (|(*RX* ^*t*^ + *μ* ^*t*^)_*i*_| − *β*/*ρ*)_+_sgn⁡[(*RX* ^*t*^ + *μ* ^*t*^)_*i*_], *i* = 1,…, *M*;
(2) update *X* by (22) with only one iteration;
(3) *μ* ^*t*+1^ = *μ* ^*t*^ + *RX* ^*t*+1^ − *V* ^*t*+1^.

## 3. Convergence

There are many convergence proofs for both constrained and unconstrained ADMM [19, 20]. Therefore, we only need to limit ourselves to discussing the convergence of the *X*-update. We will theoretically prove that the iteration sequence will converge to a global solution if we use it to pursue an accurate solution without considering the computational cost. In the appendix, we will prove that update (22) can iteratively and monotonically minimize the cost function (10) while observing the nonnegativity constraint along the lines of [35–39].

## 4. Experiments

### 4.1. Simulated Head Phantom Data

**Figure 1:**
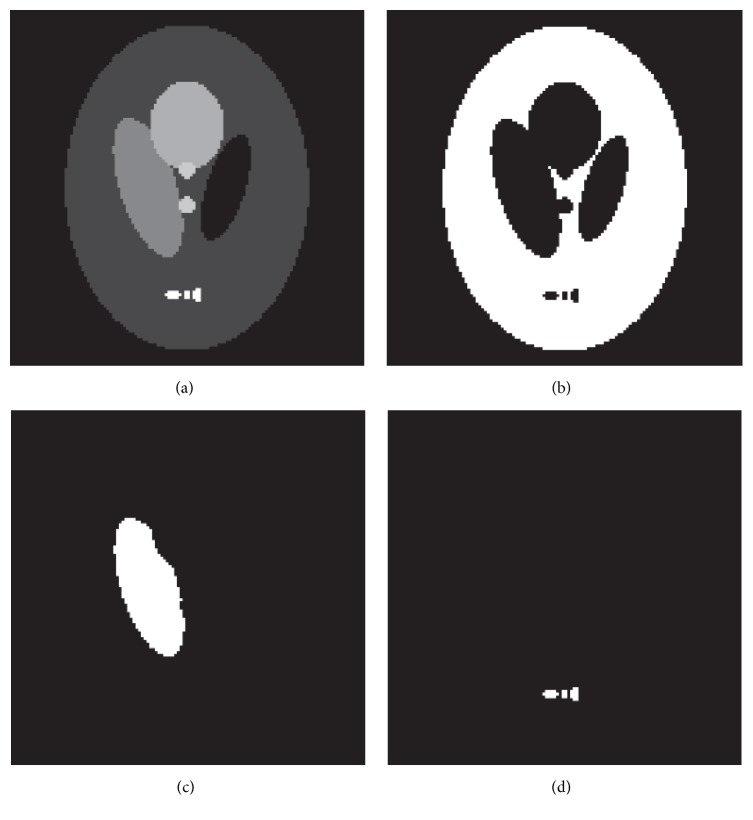
Shepp-Logan phantom with 128 × 128 grids: (a) Shepp-Logan phantom, (b) background, (c) low-activity ROI, and (d) high-activity ROI.

A simulated head phantom with 128 × 128 pixels (pixel width of 4 mm), as shown in Figure 1, is used in the following experiments. This phantom is modified to meet the needs of PET simulation because the original one is a CT phantom. There are many advantages to using simulated phantoms, including prior knowledge of the pixel values and the ability to control noise. The total detection counts are approximately 5 × 10^5^.

An anisotropic TV regularization is used to test the algorithmic performance as follows:

(23) $$\begin{array}{c} G\left(\;X\;\right)=\overset{N}{\underset{j=1}{\sum }}\left(\;\left|\;X_{j}-X_{j,\text{right}}\;\right|+\left|\;X_{j}-X_{j,\text{below}}\;\right|\;\right), \end{array}$$

where *X*_*j*,right_ and *X*_*j*,below_, respectively, represent pixels to the right and below *X*_*j*_.

We compare the performance of the proposed method with De Pierro's ISRA (image space reconstruction method) [34] and the PWLS-EM algorithm with a quadratic smoothing regularization [11]. These methods are selected to demonstrate the difference between regularized and nonregularized reconstruction algorithms. Moreover, the difference between *L*_1_-norm regularization and squared *L*_2_-norm regularization is examined. We also use several methods to pursue *X*-update, including the projected Landweber and the conjugate gradient methods. The projected Landweber method uses *α* = 1/[‖*P*‖_1_‖*P*‖_*∞*_ + *β*‖*R*‖_1_‖*R*‖_*∞*_] [40], where ‖·‖_1_ and ‖·‖_*∞*_ denote the 1- and *∞*-norms of a matrix, respectively. The code of the conjugate gradient method comes from [41], which is slightly modified to meet our criteria.

In the following, the results of the ADMM-type algorithms are named ADMM-*x*-*y*-*z*, where *x* represents the *X*-update method, which can be EM (proposed), PL (projected Landweber), and CG (conjugate gradient); *y* denotes the number of outer loops; and *z* is the number of inner loops. For example, ADMM-EM-400-120 refers to using the proposed method to update *X* with 120 inner iterations and update all of *V*, *X*, and *μ* with 400 outer iterations. In the unambiguous case, we will omit *x*, *y*, *z*, or all of them for notational simplicity. The experiments are performed on a HP Compaq PC with a 3.00 GHz Core i5 CPU and 4 GB of memory. The algorithms are implemented in MATLAB 7.0. All of the algorithms are initiated using the same uniform image for a fair comparison.

The system matrix is obtained using the “angle of view” method [28]. The diagonal matrix Σ is computed using Fessler's “data-plugin” technique [1].

Using the system matrix, we project the phantom on the sinogram with 128 radial bins (bin size of 4 mm) and 128 angular views evenly spaced over *π*. The noisy projections are obtained by Fessler's pseudorandom formulation.

(24) yi=ciPoissonci−1yi∗+aiyi∗−ciPoissonci−1aiyi∗,

where *y*_*i*_^*∗*^ is the noise-free projection, *a*_*i*_ = 30% simulates the contribution of random events, and *c*_*i*_ is the *i*th detector efficiency. We select *c*_*i*_ = 1 and thus ignore the influence of the detector efficiency. Furthermore, we ignore *S* in (2) during the simulation process.

Mean absolute error (MAE) can be used to measure the proximity of the reconstructed image to the true image. The MAE value is calculated by taking the average of the absolute difference between the reconstructed pixel values and the real ones over the entire image. The best algorithm will provide the lowest MAE value.

(25) $$\begin{array}{c} \text{MAE}\left(\;t\;\right)=\frac{1}{N}{\left\|\;X^{t}-X^{\text{True}}\;\right\|}_{1}. \end{array}$$

The following criterion is applied to stop the iteration process:

(26) $$\begin{array}{c} \chi \left(\;t\;\right)=\frac{{\left\|X^{t+1}-X^{t}\right\|}^{2}}{{\left\|X^{t}\right\|}^{2}}<\epsilon , \end{array}$$

where *ϵ* is a difference tolerance.

Contrast and variability are typically used to evaluate image quality. We compute these parameters using the method of NEMA [42]. Denote mean_*Ω*_(*X*) and std_*Ω*_(*X*) as the mean and standard deviation of the image *X* on the region *Ω*; then

(27) Contrast=meanΩ1X/meanΩ2XmeanΩ1XTrue/meanΩ2XTrueVariability=stdΩ1XmeanΩ1X,

where *Ω*_1_ represents the ROI (region of interest) and *Ω*_2_ denotes the background.

The running time of the algorithms is easily influenced by many factors, for example, coding level and running environment. Therefore, we also compare the computational complexity for the sake of fairness. Here, we use a simple and feasible method by counting the number of multiplications of the system matrix and any vector because the TV matrix (*R*) is very sparse, and thus the computation load can be ignored. In fact, for all of ADMM-PL, ADMM-CG, and ADMM-EM, each inner loop (*X*-update) requires only two multiplications of the system matrix and a vector; consequently, the computation complexity can be further simplified to the total number of inner loops, which equals the multiplications of the number of outer loops and inner loops for each *X*-update. In the following, we will take it to indicate the computational complexity.

**Figure 2:**
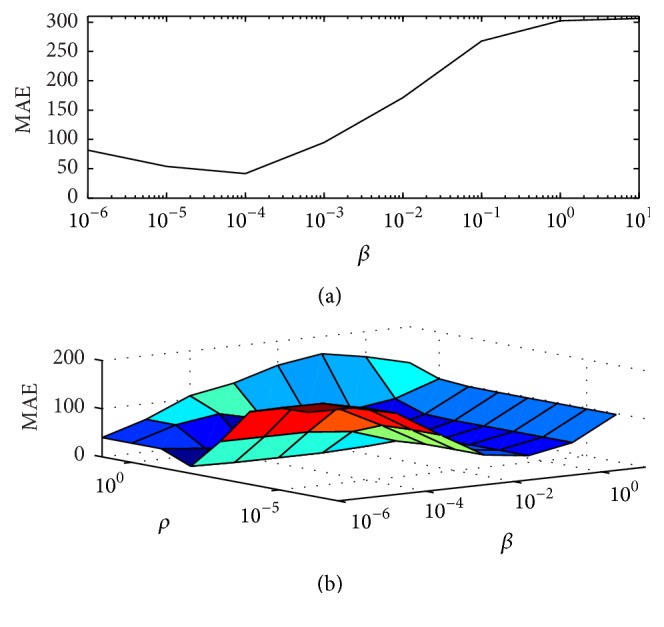
MAE values generated by the greedy enumeration strategy: (a) PWLS-EM with 400 iterations and (b) ADMM-EM with 400 outer iterations and 120 inner iterations. The global minimum MAE values correspond to the optimal penalty parameters. Note that both the *x*- and *y*-axes are log-scale to provide a clear visualization. PWLS-EM obtains the minimal MAE value (41.91) at *β* = 10^−4^, and ADMM-EM obtains the minimal value (13.05) at (*β*, *ρ*) = (10^−2^, 10^−4^).

In Figure 2, we find a suitable penalty parameter for PWLS-EM by comparing the change of the MAE at the 400th iteration with respect to *β*. We also pursue the optimal *β* and *ρ* for ADMM-EM by comparing the change of MAE at the 400th outer iteration and the 120th inner iteration. Since the *X*-update by EM can be proven in theory to converge to a single global optimum, ADMM-EM may be viewed as a golden standard. For PWLS-EM, the optimal MAE values are obtained at *β* = 10^−4^. For ADMM-EM, the optimal penalty parameter is obtained at (*β*, *ρ*) = (10^−2^, 10^−4^). In addition, the minimal MAE values for PWLS-EM and ADMM-EM are 41.91 and 13.05, respectively. This phenomenon shows a clear advantage of *L*_1_-norm regularization compared to Tikhonov-type regularization.

**Figure 3:**
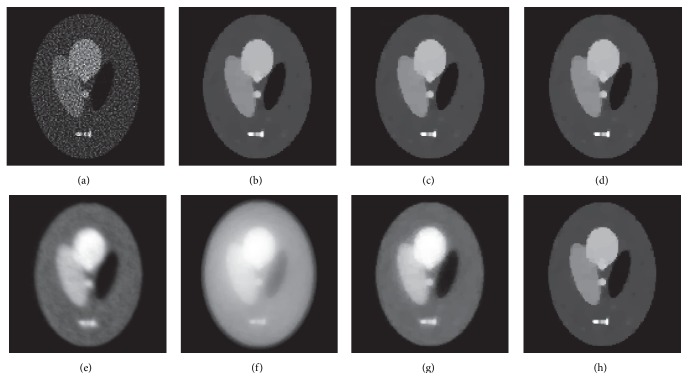
Reconstructed images by (a) ISRA with 400 iterations, (b) ADMM-PL-400-120, (c) ADMM-CG-400-120, (d) ADMM-EM-400-120, (e) PWLS-EM with 400 iterations, (f) ADMM-PL-400-1, (g) ADMM-CG-400-1, and (h) ADMM-EM-400-1.

Below, we focus on several special algorithms, including ISRA, PWLS-EM (*β* = 10^−4^), and ADMM (*β* = 10^−2^ and *ρ* = 10^−4^).  Figure 3 shows the reconstructions corresponding to a greedy outer and inner iteration. As shown in this figure, ISRA's image suffers from serious noise artifacts, and PWLS-EM fails to preserve the edges. ADMM-PL-400-1 and ADMM-CG-400-1 provide an obvious blurred reconstruction. The remaining images exhibit smooth interiors and sharp edges, which are desirable results. These algorithms provide almost identical reconstructed results. In fact, since the subproblem to update the pixel values has a strictly convex cost function, every convergent algorithm will converge to the same result with a greedy iteration number. This is the reason for why ADMM-PL-400-120, ADMM-CG-400-120, and ADMM-EM-400-120 provide similar results. This experiment also indicates that EM is more suitable for the simplified ADMM algorithm than PL and CG. We believe that the reason is because the EM-*X*-update can ensure the monotonic decrease of the augmented Lagrangian function; however, the projected Landweber and CG have no such characteristic.

**Figure 4:**
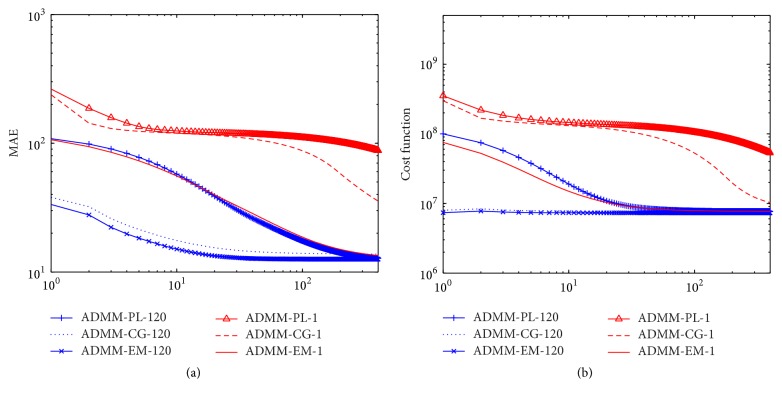
MAE and cost function versus the number of outer loops, where the greedy versions of ADMM use 120 iterations for the inner loop to update image pixels: (a) MAE and (b) cost function.

Figure 4 presents a comparison of the MAE curves and cost function with increasing iteration numbers for ADMM-PL, ADMM-CG, and ADMM-EM, in which the curves of ISRA and PWLS-EM disappear because they clearly fail to preserve edges and suppress noise artifacts. As shown, for both curves, ADMM-PL-1 presents larger MAE and cost function values and ADMM-CG-1 provides the next-worse curve, demonstrating the consistent conclusion that ADMM-PL and ADMM-CG are ill-suited for the proposed simplified strategy. ADMM-EM-120 exhibits the fastest convergence rate; however, it is only slightly superior to ADMM-CG-120. ADMM-PL-120 and ADMM-EM-1 present similar curves. In conclusion, ADMM-EM-120 and ADMM-CG-120 perform better than ADMM-PL-120 and ADMM-EM-1 when only judging these curves, but eventually, all algorithms will approach the same target with increasing iteration numbers.

Note that ADMM-EM has been proven in theory to converge to a unique global solution of the corresponding subproblem with a greedy *X*-update. This means that when ignoring rounding error, our algorithm can arrive at any desired accuracy given a large enough iteration count. Thus, in these experiments, ADMM-PL and ADMM-CG will also converge to that solution if they are indeed convergent. However, to date, there is no evidence to show the convergence when negative values are truncated during the iteration process.

Table 1 presents the comparison of algorithmic performance, including MAE, cost function, contrast, variability, running time (second), and computational complexity. We only compare the greedy versions of ADMM-PL, ADMM-CG, and ADMM-EM with the simplified version of ADMM-EM because the simplified versions of ADMM-PL and ADMM-CG failed to obtain an acceptable image. Our findings show that either the greedy or simplified version of ADMM-EM always provides the best results. In addition, the greedy versions of ADMM with *X*-update by PL, CG, and EM impose a similar time cost and have the same computational complexity, but the simplified version of ADMM-EM requires the least number of iterations.

**Figure 5:**
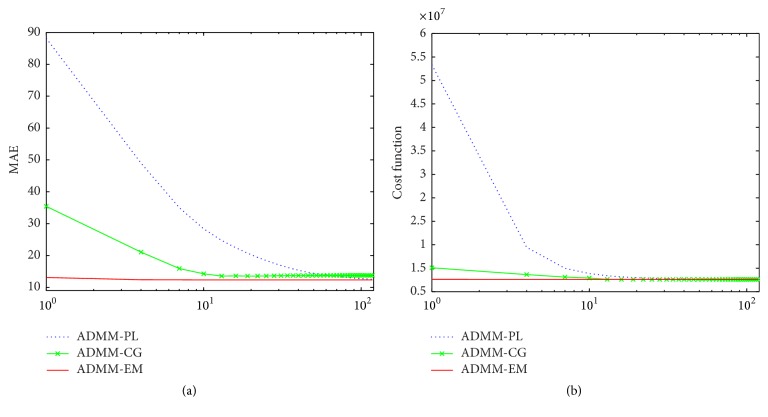
MAE and cost function versus the number of inner loops, where the number of outer loops is fixed to 400 for a sufficient convergence: (a) MAE and (b) cost function. Note that the samples of the iteration number of inner loop are collected every 3 iterations during the experiment.

Figure 5 differs from Figure 4 because the former fixes the number of inner iterations and the latter fixes the number of outer iterations. This figure also presents a comparison of MAE curves and cost function. As shown, ADMM-EM always presents the best result, ADMM-CG provides the next best result, and ADMM-PL shows the worst result; however, all of them will be consistent after sufficient inner iterations. We can also observe that ADMM-EM's result does not actually depend on the number of inner iterations, which demonstrates a clear advantage of the simplified version over the other two.

The above experiments provide a comparison while fixing the number of outer or inner iterations. In practical applications, we generally do not require this many iterations to obtain an acceptable image. In Table 2, (26) will be used as a stopping condition for both cases; then, we obtain the acceptable images with *ϵ* = 10^−8^. As shown, when fixing the number of inner iterations, the greedy ADMM-EM requires a lower number of outer iterations, whereas the simplified ADMM-EM requires more outer iterations. When fixing the outer iteration count, ADMM-EM still requires the lowest number of inner iterations, while ADMM-PL requires the most.

Table 3 also presents a comparison of algorithmic performance; however, the number of inner and outer iterations use those suggested by Table 2 to meet the stop rule *ϵ* = 10^−8^. ADMM-EM-68-11 is also included in the comparison. All presets present comparable evaluation parameters, but the simplified ADMM-EM requires very little running time and computational complexity. We observe that either the greedy or simplified version of ADMM-EM still provides the best results. In addition, the greedy version of ADMM-PL requires the largest amount of time and computational cost. The greedy versions of ADMM-EM and ADMM-CG have a similar computational cost, but the simplified version of ADMM-EM requires less computational cost. In fact, ADMM-EM-256-1 consumes 42.98% of the running time of ADMM-CG-71-11 and 9.37% of that of ADMM-PL-292-20, which corresponds to 32.78% and 4.38% of computation complexity, respectively.

### 4.2. PET Clinical Data

**Figure 6:**
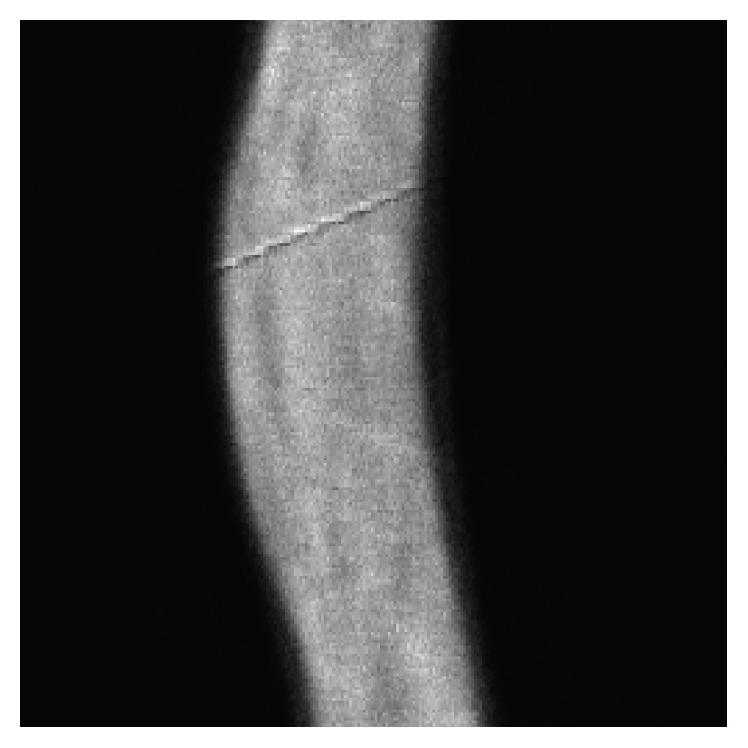
Real clinical brain projections with 128 bins and 128 angles that are acquired from a mPower scanner.

Real clinical brain projections, which were obtained using Positron's mPower scanner with 10 *μ*Ci FDG preinjected into a patient, were also used for evaluation purposes. The acquisition lasted for 10 minutes, and 700 M counts in 61 slices were obtained. The normalization was performed by measurements obtained from a calibration scan with a 1 *μ*Ci Ge^68^ rotating rod source. The attenuation coefficients of the attenuation media were computed from a transmission scan of a 5 *μ*Ci Ge^68^ rod source. The raw data were 128 radial bins and 128 angles (bin size 4 mm), which are shown in Figure 6. The ADMM-type algorithms with (*β*, *ρ*) = (10^−2^, 10^−4^) obtain the best reconstruction as above, which we also use on the real clinical data. We also executed the proposed iteration numbers in Table 3 for the algorithms.

**Figure 7:**
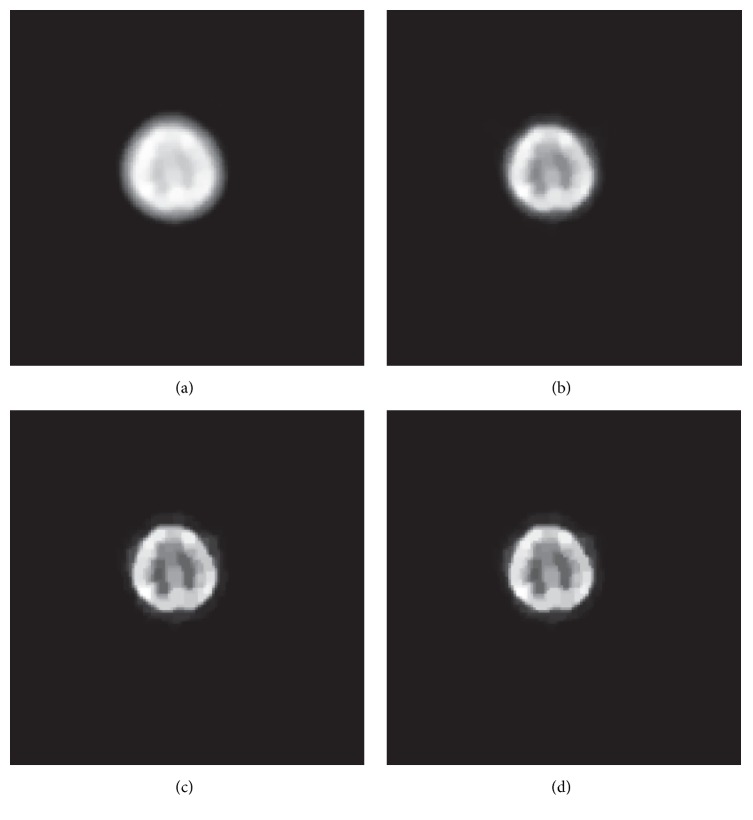
The reconstructed results of real clinical brain PET data are, respectively, obtained by (a) PL-292-20, (b) CG-71-11, (c) EM-68-11, and (d) EM-256-1. Every result is scaled according to its own minimum and maximum.

Figure 7 shows the reconstructions of the patient's projection data. For real clinical data, it is difficult to quantitatively evaluate the algorithms. The reconstructed grid is 128 × 128, with 4 mm pixels. This figure illustrates that the two proposed algorithms produce sharper images with greater contrast than those achieved with other algorithms. Moreover, PL-292-20 and CG-71-11 do not clearly resolve the ellipse. Among them, PL-292-20 results in a seriously degraded image, where the boundaries of the different regions are obscure. The large difference is due to the lower number of iterations. These experimental results further demonstrate that ADMM-EM leads to a superior result than the others by providing a fast convergence.

From the derivation of the proposed method, we make the following observation: the EM-type *X*-update ensures that the cost function decreases at each iteration. However, it is difficult for the other algorithms to meet this condition, particularly for lower numbers of iterations. Thus, ADMM-EM with one inner loop can achieve a good result. Certainly, while running many inner loops, all of the algorithms will converge to the same solution; thus, they present some similar images.

## 5. Conclusion

We present a special application of ADMM to PET image reconstruction. Specifically, a new update rule is developed to iteratively update the pixel values, which exhibits desirable properties, including monotonic decrease of the cost function, self-constraining to the feasible region, and no need to impose a step size. Such properties allow us to implement a simplified version of ADMM, requiring considerably less time and computational overhead. We provide a rigorous theoretical global convergence proof for the update step. The simulation results demonstrate that the proposed greedy algorithm provides a stabler and faster convergence with similar computational cost as ADMM-PL and ADMM-CG. The results also indicate that the proposed simplified algorithm obtains a similar image quality while imposing lower computational costs.

For our simplified algorithm, a theoretical convergence proof cannot be provided; rather, we use the experimental method to demonstrate the convergence. Proving the convergence for the simplified algorithm will be the focus of future work.

## Figures and Tables

**Table 1:** Algorithmic performance including mean absolute error (MAE), cost function (Fun.), contrast (LC and HC denote that of low and high ROI, resp.), variability (Var.), running time (Time, units of seconds), and computational complexity (Comp.), in which we use to represent the best value. All experiments are executed twenty times to obtain an average value.

ADMM-	MAE	Fun. (10^6^)	LC (%)	HC (%)	Var. (%)	Time	Comp.
PL-50-10	82.40	44.55	71.74	33.85	12.56	6.64	500
CG-50-10	16.00	7.70	97.48	78.06	11.26	10.79	500
EM-50-10	[13.73]	[7.67]	98.33	[83.44]	[10.91]	6.71	500
EM-50-1	25.69	8.24	[98.54]	74.93	13.66	[1.37]	[50]

PL-100-20	42.88	12.21	89.98	48.17	15.62	25.35	2000
CG-100-20	14.26	[7.66]	98.01	83.18	10.73	33.56	2000
EM-100-20	[13.12]	[7.66]	98.15	[84.09]	[10.69]	25.27	2000
EM-100-1	18.98	7.84	[98.61]	81.17	12.27	[2.73]	[100]

PL-200-40	20.47	7.99	96.74	68.93	12.65	99.47	8000
CG-200-40	14.22	7.65	98.24	83.82	10.73	115.17	8000
EM-200-40	[13.03]	[7.64]	98.32	[84.35]	[10.68]	97.62	8000
EM-200-1	15.30	7.69	[98.63]	83.77	11.34	[5.42]	[200]

PL-300-80	14.43	7.68	97.98	81.03	11.13	295.93	24000
CG-300-80	14.20	7.65	98.24	84.30	10.69	318.08	24000
EM-300-80	[12.96]	[7.64]	98.31	[84.78]	[10.64]	288.76	24000
EM-300-1	14.23	7.68	[98.54]	84.66	11.02	[8.14]	[300]

PL-400-120	13.23	7.65	98.26	83.85	10.87	590.77	48000
CG-400-120	14.23	[7.64]	98.19	83.84	10.82	617.80	48000
EM-400-120	[13.05]	[7.64]	98.26	84.28	[10.78]	615.27	48000
EM-400-1	13.15	7.66	[98.42]	[84.30]	10.88	[10.84]	[400]

**Table 2:** Inner and outer iteration numbers to meet *ϵ* = 10^−8^ in (26). For Case 1, we fix 120 inner iterations for the greedy ADMM algorithms; for Case 2, we fix 400 outer iterations.

Algorithm	Iteration number
Case 1: given inner iteration number	Outer iteration number

ADMM-PL-120	292
ADMM-CG-120	71
ADMM-EM-120	68
ADMM-EM-1	256

Case 2: given outer iteration number	Inner iteration number

ADMM-PL-400	20
ADMM-CG-400	11
ADMM-EM-400	1

**Table 3:** Comparison of algorithmic performance similar to Table 1. We still use [ ] to represent the best value, and all experiments are still executed twenty times to obtain an average value. Note that ADMM-EM-68-11 uses the same inner iteration count as ADMM-CG.

ADMM-	MAE	Fun. (10^6^)	LC (%)	HC (%)	Var. (%)	Time	Comp.
PL-292-20	23.31	8.31	96.11	64.37	13.25	74.17	5840
CG-71-11	14.73	7.70	97.85	81.21	10.78	16.17	781
EM-68-11	[13.21]	[7.67]	98.42	[84.52]	[10.61]	9.94	748
EM-256-1	14.45	7.68	[98.62]	84.33	11.00	[6.95]	[256]

## Appendix

In theory, the Karush-Kuhn-Tucker (KKT) point will be a global solution if the cost function is convex. It is easy to see the convexity of Φ(*X*). By Theorem  2.19 in [43], the KKT conditions of (10) are as follows:

(A.1) ∂ΦX∂Xj=0if  Xj>0

(A.2) ∂ΦX∂Xj≥0if  Xj=0.

In the following, we use a number of reasonable assumptions.

Assumption A.1 A.1. For the iteration sequence {*X* ^*t*^}, we assume that

(1) the algorithm starts from a positive image;
(2) (*P* ^*T*^Σ*P*)_*jj*_ > 0 for all *j*;
(3) Φ is a strictly convex function.

The first assumption forces the iterations to be positive, but the limit may be zero. The second condition is reasonable because (*P* ^*T*^Σ*P*)_*jj*_ = 0 suggests that *P*_*ij*_ = 0 for any *i*. Thus, the equation *Y* = *PX* is irrelevant to *X*_*j*_, and then *X*_*j*_ in *X* is removable. For the third requirement, one of the main reasons to add the regularization is to enforce the convexity.

First, we provide four useful lemmas.

Lemma A.2 A.2. The set of accumulation points of a bounded sequence {*Z* ^*t*^} with {‖*Z* ^*t*+1^ − *Z* ^*t*^‖} → 0 is connected and compact.

Proof See Theorem 28.1, Ostrowski [44].

Lemma A.3 A.3. The iteration sequence {*X* ^*t*^} is bounded.

Proof We have {*X* ^*t*^}⊂{*X* : Φ(*X*) ≤ Φ(*X* ^0^)}⊂{*X* : *F*(*X*) ≤ Φ(*X* ^0^)}; therefore, if *F* has bounded level sets, {*X* ^*t*^} is bounded. The boundedness of the level sets of *F* can be proven by contradiction. Otherwise, for a given real value $\hat{Z}$, there must exist a sequence {*Z* ^*t*^} that satisfies $F(Z^{t})\leq \hat{Z}$ and an element {*Z*_*j*_^*t*^} that approaches +*∞*. Considering that *P*_*ij*_ ≥ 0 and ∑_*i*=1_^*N*^ *P*_*ij*_^2^ > 0 (Assumption A.1) for all *j*, then it implies that *F*(*Z* ^*t*^)→+*∞*, which is a contradiction.

Lemma A.4 A.4. The sequence {‖*X* ^*t*^ − *X* ^*t*+1^‖} → 0. Furthermore, if a subsequence {*X* ^*t*_*s*_^} → *X* ^*∗*^, then {*X* ^*t*_*s*_+1^} → *X* ^*∗*^ as well.

Proof (1) Take into account that

(A.3) ∇jj2ϕX ∣ Xt=2PTΣPXt+SjXjt+2ρR¯TR¯Xt+C¯jXjt+R^TR^Xt+C^jXjt≥2PTΣPXtjXjt≥2PTΣPjj.

Let *γ* = min_*j*_{(*P* ^*T*^Σ*P*)_*jj*_}. Since ∇*ϕ*(*X* ^*t*+1^∣*X* ^*t*^) = 0, then

(A.4) ΦXt−ΦXt+1≥ϕXt ∣ Xt−ϕXt+1 ∣ Xt=12Xt−Xt+1T∇2ϕXt+1 ∣ XtXt−Xt+1≥γXt−Xt+12.

Because {Φ(*X* ^*t*^)} monotonically decreases and it is bounded from below, then {Φ(*X* ^*t*^) − Φ(*X* ^*t*+1^)} → 0; thus, {‖*X* ^*t*^ − *X* ^*t*+1^‖} → 0. (2) By contradiction, if {*X* ^*t*_*s*_+1^} diverges, then it must have a convergent subsequence {*X* ^*t*_*s*__*s*_+1^} → *X* ^*∗∗*^ ≠ *X* ^*∗*^ because of the boundedness by Lemma A.3. Let *ϵ*_0_ = ‖*X* ^*∗*^ − *X* ^*∗∗*^‖ > 0. Consider the two convergent subsequences {*X* ^*t*_*s*__*s*_^} and {*X* ^*t*_*s*__*s*_+1^}; then, there must be a positive integer *T* to make ‖*X* ^*t*_*s*__*s*_^ − *X* ^*∗*^‖ < *ϵ*_0_/4 and ‖*X* ^*t*_*s*__*s*_+1^ − *X* ^*∗∗*^‖ < *ϵ*_0_/4 when *t*_*s*_*s*__ > *T*. By the triangle inequality, we can obtain the contradictive result to {‖*X* ^*t*^ − *X* ^*t*+1^‖} → 0 as

(A.5) Xtss−Xtss+1+Xtss−X∗+Xtss+1−X∗∗≥X∗−X∗∗⟹Xtss−Xtss+1>ϵ02.

Lemma A.5 A.5. At each iteration, one knows that

(A.6) Xjt+1=Xjt−αj∇ΦXtj,where  αj=Xjt2A3j+2ρA4j.

The derivation process is omitted due to its simplicity. By the three theorems below, the global convergence will be proven.

Theorem A.6 A.6. Let {*X* ^*t*_*s*_^} → *X* ^*∗*^ be any convergent subsequence; then *X* ^*∗*^ meets the first KKT condition (A.1).

Proof When *X*_*j*_^*∗*^ > 0, by (21), it can be verified that

(A.7) ∂fX ∣ X∗∂XjX∗=2PTPX∗−Y+Sj∂g¯¯X ∣ X∗∂XjX∗=2R¯TR¯X∗+C¯−2R¯TR^X∗+C^∂g^^X ∣ X∗∂XjX∗=2R^TR^X∗+C^−2R^TR¯X∗+C¯.

Then ∂*ϕ*(*X*∣*X* ^*∗*^)/∂*X*_*j*_|_*X*^*∗*^_ = ∂Φ(*X* ^*∗*^)/∂*X*_*j*_. We consider that ∂*ϕ*(*X*∣*X* ^*t*_*s*_^)/∂*X*_*j*_|_*X*^*t*_*s*_+1^_ = 0. Because {*X* ^*t*_*s*_^} → *X* ^*∗*^ and {*X* ^*t*_*s*_+1^} → *X* ^*∗*^ (Lemma A.4), then

(A.8) $$\begin{array}{c} {\left.\;\frac{\partial \phi \left(X{\;|\;X}^{*}\right)}{\partial X_{j}}\;\right|}_{X^{*}}=\underset{t_{s}\to +\infty }{\lim}{\left.\;\frac{\partial \phi \left(X\;|\;X_{s}^{t}\right)}{\partial X_{j}}\;\right|}_{X^{t_{s}+1}}=0. \end{array}$$

Theorem A.7 A.7. The entire sequence {*X* ^*t*^} converges.

Proof According to Lemmas A.2, A.3, and A.4, the set of accumulation points of {*X* ^*t*^} is connected and compact. If we can prove that the number of accumulation points is finite, then the desired result follows because a finite set can be connected only if it consists of a single point [38]. To prove the existence of a finite number of accumulation points, we consider any accumulation point *X* ^*∗*^. Given an integer set *Ω* = {1,2,…, *T*}, where *T* is the total number of components of *X*, then *Ω* ^*∗*^ = {*j* : *X*_*j*_^*∗*^ = 0} is a subset of *Ω*. Let Φ_*Ω*^*∗*^_ be the restrictions of Φ to the set {*X* : *X*_*j*_ = 0, *j* ∈ *Ω* ^*∗*^}, which is a strictly convex function of the reduced variables. It follows that Φ_*Ω*^*∗*^_ has a unique minimum (Theorem A.6: ∂Φ(*X* ^*∗*^)/∂*X*_*j*_ = 0  if  *X*_*j*_^*∗*^ > 0). It means that an accumulation point must correspond to a subset of *Ω*. The number of subsets of *Ω* is finite; thus, the number of accumulation points is also finite.

In Theorem A.6, we prove that every accumulation point meets the first KKT condition, by which the full sequence convergence is provided in Theorem A.7. Naturally, the limit of {*X* ^*t*^} satisfies the first KKT condition. In the following, we will show that the second KKT condition is also satisfied.

Theorem A.8 A.8. The limit *X* ^*∗*^ of {*X* ^*t*^} meets the second KKT condition (A.2).

Proof When *X*_*j*_^*∗*^ = 0, by contradiction, we assume that there is *X*_*j*_^*∗*^ = 0 satisfying [∇Φ(*X* ^*∗*^)]_*j*_ < 0. Since {*X* ^*t*^} → *X* ^*∗*^, there exists *ϵ* < 0 and a positive integer *𝒯* such that [∇Φ(*X* ^*t*^)]_*j*_ < *ϵ* for *t* > *𝒯*; then

(A.9) Xjt+1−Xjt=−αj∇ΦXtj>−αjϵ>0by  Lemma  A.5.

Thus, we can obtain that *X*_*j*_^*t*+1^ > *X*_*j*_^*t*^, which is a contradiction to {*X*_*j*_^*t*^} → 0.
